# Strategies for improving the reporting of human immunophenotypes by flow cytometry

**DOI:** 10.1186/2051-1426-2-18

**Published:** 2014-06-18

**Authors:** Michael P Gustafson, Yi Lin, Mabel Ryder, Allan B Dietz

**Affiliations:** 1 Department of Laboratory Medicine and Pathology, Mayo Clinic, 200 1st St. SW, Rochester, MN 55905, USA; 2 Division of Hematology, Mayo Clinic, 200 1st St. SW, Rochester, MN 55905, USA; 3 Division of Endocrinology, Mayo Clinic, 200 1st St. SW, Rochester, MN 55905, USA

**Keywords:** Flow cytometry, Immunophenotypes, Myeloid derived suppressor cells, Human immunology, Biomarkers

## Abstract

**Background:**

Flow cytometry is the gold standard for phenotyping and quantifying immune cells. New technologies have greatly increased our capacity to measure both routine and complex immunophenotypes. The reporting of immunophenotype data is not consistent in human studies yet it is quite critical for understanding disease specific changes, responses to immunotherapies, and normal immune homeostasis. Here we examine the barriers that hinder cross comparisons of flow cytometry data collected from human studies and clinical trials.

**Findings:**

We demonstrate that phenotypes reported as percentages within a cell compartment (i.e. myeloid derived suppressor cells as a percent of mononuclear cells) without providing data on the parent population may contribute to misleading conclusions. The enumeration of phenotypes as cell counts (cells/μl) provides a basis to more accurately compare the relationships among phenotypes. Finally, we provide evidence that density gradient centrifugation, which eliminates the ability to measure phenotypes as cell counts, can affect the expression of surface markers and consequently alter the distribution of particular immunophenotypes.

**Conclusions:**

We propose that by measuring immunophenotypes as cell counts from minimally manipulated samples (whole blood) will improve the reporting of flow data and facilitate more direct comparisons of data across human studies.

## Findings

### Introduction

Flow cytometry has become a foundational tool to analyze the immune system. The emergence of multiparametric analyses using novel fluorochromes has greatly expanded our ability to dynamically characterize a broad repertoire of immunophenotypes. However, careful controls and methodologies are required to generate reproducible and scientifically valid data, as was recently highlighted in a review by Maecker et al. [1]. We have identified that commonly used approaches for sample preparation (i.e. density gradient centrifugation) and the statistical analysis resulting from this approach leads to potentially erroneous data and misinterpretations about changes in immunophenotypes.

Current standard approaches in research laboratories rely on density gradient centrifugation and often followed by cryopreservation. While this step circumvents the problems of storing whole blood samples, it removes over 50% of the entire leukocyte population, namely granulocytes (basophils, eosinophils and neutrophils). The data derived from density centrifugation-prepared samples moreover relies on three critical assumptions; 1) that pathologies do not affect the density of cells, 2) that immune phenotypes are unaffected and 3) that only granulocytes are eliminated. Despite this technique being widely used, there is little (if any) supporting data demonstrating the accuracy of this isolation step nor or the purity of the isolated population. Perhaps most importantly, this method fails to permit quantification of cell counts in blood (cells/μl). As clinical flow labs have well documented, cell counts allow for more accurate and reliable comparisons across time and between labs [2-6].

The field of clinical flow cytometry has moved away from density gradient centrifugation and has benefited greatly from enumerating immune phenotypes from whole blood. There is a substantial amount of data reported in the literature demonstrating that single platform assays utilizing absolute counts can be validated for multi-institutional studies. For example, absolute CD4^+^ cell counts in HIV patients has been crucial for defining HIV/AIDS disease status and is part of the criteria for treatment decisions. Indeed, the use of flow cytometry for measuring CD4^+^ cell counts has become so routine, that most clinical studies do not report how the CD4^+^ cells were quantified [2]. Whitby et al. reported that the standardization of CD4 enumeration by single platform method reduced inter-laboratory CV to less than 5% [5]. In addition to the CD4 assay, the enumeration of CD34^+^ cells for hematopoietic stem cell transplantation has been another assay that has been very successful in harmonizing data comparison across institutions. Gratama and colleagues validated a single platform assay (ISHAGE) for measuring cells/μl and reported inter-laboratory CVs of about 10% across 36 laboratories [3]. This method was subsequently credited for being a major factor in reducing the variability of CD34 enumeration in a nine year follow up study [4]. These examples should compel us in the research community to include some of these principles to standardize assays in translational research.

Another consequence of density based purification of cells for flow analysis is that immunophenotypes can only be expressed as a fraction or percentage of a larger group. This is a very common practice, where the phenotype of interest (regulatory T cells or myeloid derived suppressor cells, for example) is reported as a percent of a larger group (% Tregs = CD4^+^CD127^low^CD25^+^/CD4^+^). Conclusions are drawn regarding the change in percentage, with assumptions that the parent or grandparent population is fixed. For example, the increases in the regulatory T cell population reported as a percent of the parent population (CD4) can change either by increasing the regulatory T cells and holding the CD4 population constant or by not changing (or even reducing) the regulatory T cell population and simultaneously reducing the CD4 population to a greater extent. In some cases, the parent population is represented via the mononuclear cells that were assumed to be purified during density centrifugation. While measuring frequencies of subsets is still valuable and useful for the development of biomarkers associated with clinical outcomes, reporting the data without additional context (i.e. data regarding the parent/grandparent population) can often result in an incomplete picture of the true relationships of one phenotype to another.

### Results and discussion

**Figure 1:**
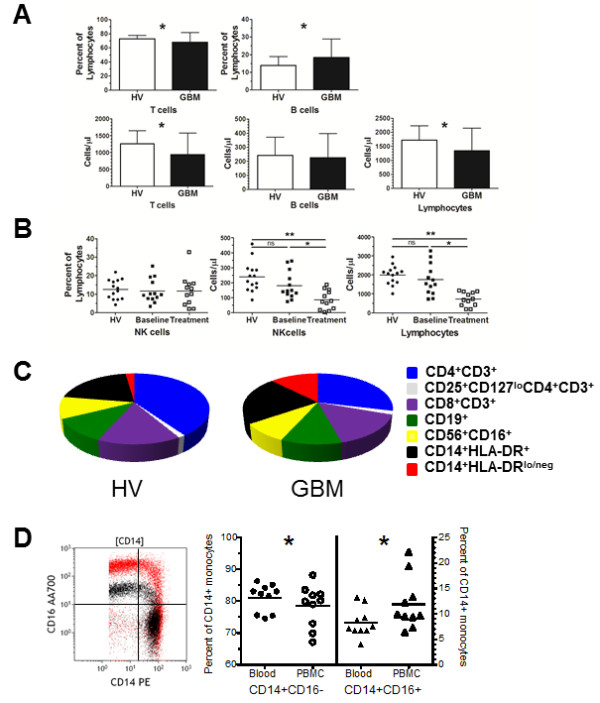
**The method of data presentation and sample preparation can affect immune phenotype results. A**. T cells, B cells, and lymphocytes were reported as a fraction of a larger population (top graphs) or enumerated by cell counts (cells/μl) (bottom graphs) from glioblastoma patients (n = 27) and healthy volunteer controls (n = 40). **B**. NK cells reported as a percentage of lymphocytes or NK cells and lymphocytes reported per unit of blood for GBM patients at baseline (n = 13), treatment (n = 12) and compared to HV controls **C**. Measuring leukocytes per unit of blood allows complete reconstitution of the composition of blood. Pie graphs representing the mononuclear compartment of healthy volunteers (HV) and glioblastoma patients (GBM) includes CD4^+^CD3^+^ T cells (dark blue), CD8^+^CD3^+^ T cells (light blue), regulatory T cells (CD25^+^CD127^lo^CD4^+^CD3^+^, white), CD19^+^ B cells (green), CD56^+^CD16^+^ NK cells (yellow), CD14^+^HLA-DR^+^ monocytes (black), and CD14^+^HLA-DR^lo/neg^ monocytes (red). **D**. CD14 and CD16 bivariate plot overlay of CD14+ gated monocytes from cells stained from whole blood (black) and the same sample with cells stained from density gradient centrifugation (PBMC; red). Comparison of classical (CD14^+^CD16^−^) and intermediate monocyte (CD14^+^CD16^+^) populations from ten healthy volunteer blood samples that were either directly stained from whole blood or stained from PBMCs. All studies were performed under the review and approval of the Mayo Clinic Institutional Review Board. Asterisk indicates p < 0.05 and ** indicates p < 0.005 by Mann–Whitney test for unpaired samples.

To illustrate the issues with sample collection and incomplete data reporting, we reanalyzed the data from several of our published studies in which we have phenotyped leukocyte populations by flow cytometry from human subjects [7-11]. Our samples were analyzed using (freshly isolated) whole blood allowing us to determine both the number of cells/μl (via single platform bead indexing) and relative percentages. In our cohort of glioblastoma patients (GBM) and healthy volunteer (HV) controls [8,9], the T cell compartment was decreased and B cell population was elevated in GBM patients compared to HV controls when measured as a percent of lymphocytes (Figure 1A). However, when the populations were compared using cells/μl only the T cells were different between GBM and HV groups. This change resulted from specific loss of T cells in GBM patients that affect the percentage of B cells when expressed as a fraction. In a separate cohort, blood samples from GBM patients enrolled in clinical trial #NCCTG N027D were collected at various times before and after treatment [11]. This trial was initiated to test whether the addition of temsirolimus (CCI-779) to chemoradiation would improve the responses to treatment versus chemoradiation alone. We performed immunophenotyping on the treated GBM patients to assess the effects of the treatment on immune cells and to determine why three patients enrolled early in the trial developed fatal infection-related toxicities. We measured lymphocyte populations by cell counts and as percentages of total lymphocytes. This treatment resulted in profound suppression of T cells, B cells, and NK cells. Here, we highlight this trial as an example of the importance of quantifying immune cells in addition to just measuring their frequency. The levels of NK cells, as measured by a percent of total lymphocytes, revealed no differences at baseline or on treatment compared to HV controls (Figure 1B). When NK cells were analyzed using cell counts, there was a reduction in NK cells following treatment. Again, the disparity between the two measurements was the result of a drop in the lymphocyte compartment after GBM patients were treated even as the ratio of NK cells to total lymphocytes did not change. Although the drop in NK cells likely reflects a general decline in the entire lymphocyte pool, had we measured these phenotypes from density purified cells, we would not have detected the profound lymphopenia and thus concluded that the treatment was not affecting the lymphocyte compartment. The conclusions drawn from this scenario could have had severe consequences as it could have put additional patients on trial at risk for infection-related toxicities. These examples underscore the significance of understanding the data within a more global context. Unifying the context (turning all data into cells/μl) permits accurate and reliable data analysis. Moreover, accounting for the entire mononuclear compartment provides a more comprehensive perspective on the entire immune system. The data can be graphed in a way to visualize either the entire leukocyte compartment or mononuclear cells. Figure 1C shows the average mononuclear compartment of 40 healthy volunteers compared to the average of 27 GBM patients [8] and demonstrates how the subsets change in a particular disease setting. Indeed, our analysis of over 25 GBM patients has demonstrated that GBM patients are lymphopenic and have elevated CD14^+^HLA-DR^lo/neg^ monocytes [8,9]. Enumeration allows comparison of relationships of previously compared phenotypes (such as CD4 and CD8 within the CD3 compartment) as well as relationships previously not evaluated (such as B cells to NK cells).

We also looked at the consistency of reporting of myeloid derived suppressor cells (MDSCs), a heterogeneous population that is of very high interest. Using PubMed, we searched the terms “human” and “myeloid derived suppressor cells” for the year “2013” (through 10/01/2013), and found 95 publications of which 13 reported data on MDSCs in peripheral blood. Of these, 10 of 13 used PBMCs as the source material and only one clearly measured the MDSC population in cell counts with corresponding parent enumeration. The majority reported MDSCs as a percent of PBMCs with the assumption again, that the parent population was constant. This is particularly problematic in leukemias like chronic lymphocytic leukemia, in which the PBMC compartment is vastly enlarged by cancerous B cells and so any reporting of MDSCs as a percent of PBMCs is rather meaningless. For example, by measuring cell counts in addition to phenotype frequency (percent of a parent group), we were able to identify significant clinical correlations between CD14^+^HLA-DR^lo/neg^ monocytes, total monocytes and disease progression in CLL [7]. In other cancers where the PBMC compartment is often smaller than the control group, MDSC reporting is further complicated by assumptions that granulocytes are completely removed upon density gradient purification. Raychaudhuri et al. show that, in their analysis of density gradient purified mononuclear cells from GBM patients, 82% of the MDSC population (as defined by CD33^+^HLA-DR^−^) were positive for the granulocytic marker CD15^+^[12]. These cells were deemed neutrophilic MDSCs. However, since granulocytes are typically classified as CD33^+^HLA-DR^−^CD15^+^, there is an obligation to demonstrate that these cells that escaped density gradient purification are indeed phenotypically and/or functionally different than regular granulocytes.

In a study that directly compares the values of MDSCs and how they change with various sample processing methods, Duffy and colleagues identify that CD14^+^HLA-DR^lo/neg^ monocytes (monocytic MDSCs) are elevated in patients with gastrointestinal cancer [13]. Elevated CD14^+^HLA-DR^lo/neg^ monocytes were observed across three different processing methods (cell counts from whole blood, density gradient purified PBMCs, and cryopreserved/thawed PBMCs). However, the authors noted that CD14^+^HLA-DR^lo/neg^ monocyte cell counts from patients differed between whole blood and fresh PBMCs by almost 2-fold, and the frequency of CD14^+^HLA-DR^lo/neg^ monocytes between cryopreserved PBMCs and fresh PBMCs was different by 2 fold. As such, the various processing steps had a large impact on the values of this cell phenotype. We support these attempts to understand the effect of sample processing has on the data and to report the whole data so that the audience can understand the changes as well.

In order to gain better comprehensive understanding of immune phenotype data from flow cytometry, we strongly suggest that, when possible, 1) unfractionated whole blood be directly stained in a manner that enables measurements of absolute cell counts and 2) if immunophenotypes are to be reported as percentages, that quantitative data on the parent or grandparent population be reported as well. This will require direct antibody staining of peripheral blood samples with the addition of counting beads. Most flow cytometers are capable of analyzing lyse/no wash samples that utilize fluorescent beads to enumerate cell populations. Multiple protocols to enumerate peripheral blood populations are now available [14-16]. If these methods are followed, the data from flow cytometry can be analyzed in new and interesting ways. This numerically defined and standardized data will permit bioinformatics approaches to identify distinct immune profiles among cancer patients [8]. This method will also permit comparisons between immune phenotypes across a spectrum of disease states. This approach does require laboratories to stain fresh samples, a situation not always amenable in multi-institution clinical trials. In these cases and/or when purifying and cryopreserving PBMCs are the best option, we recommend that steps are taken, similarly to the study by Duffy et al. [13], to understand the changes of leukocyte populations in both control and patient samples resulting from the purification and cryopreservation methods. For example, at least a subset of patients should be phenotyped before and after the isolation step in order to determine how the isolation alters the data within a patient pool. We observed for example that after staining whole blood and PBMCs from 10 healthy volunteers, there was a sizeable increase in the expression of CD16, (mean fluorescence intensity, as shown in the bivariate plot overlay in Figure 1D) that in turn influenced the reporting of classical (CD14^+^CD16^−^), intermediate (CD14^+^CD16^+^) (Figure 1D) and non-classical monocyte populations (CD14^lo^CD16^+^) (data not shown). The increase in CD16 in purified samples resulted in a higher percentage of intermediate monocytes even as CD14 was relatively unaffected by the processing. This data suggests that there may be markers that are more sensitive to processing steps and provides another example of how sample processing impacts the data.

### Conclusions

In summary, there is a clear disparity between the data reported from samples collected using density centrifugation and those without. The inconsistencies of sample preparation result in data that is difficult (if not impossible) to compare. The manipulation of the blood prior to flow cytometry and the impact of this step on the analysis and subsequent reporting of the data must be carefully reconsidered. Clinical flow cytometry has been very successful in incorporating whole blood cell enumeration assays suitable for multi-institutional studies. We propose that by incorporating steps to unify data collection and interpretation, the research data will have more translational impact to clinical research and practice.

## Abbreviations

CLL: Chronic lymphocytic leukemia; GBM: Glioblastoma multiforme; NHL: Non-Hodgkin lymphoma; MDSC: Myeloid derived suppressor cells; PBMC: Peripheral blood mononuclear cells.

## Competing interests

The authors declare that they have no competing interests.

## Author’s contributions

MG, YL, and AD contributed to the conception and design of the study. MG, YL, and AD contributed to the acquisition, analysis, and interpretation of the data. MG drafted the manuscript. MR provided critical input in revising the manuscript. All authors read and approved of the manuscript.
